# Air Quality and Exercise-Related Health Benefits from Reduced Car Travel in the Midwestern United States

**DOI:** 10.1289/ehp.1103440

**Published:** 2011-11-02

**Authors:** Maggie L. Grabow, Scott N. Spak, Tracey Holloway, Brian Stone, Adam C. Mednick, Jonathan A. Patz

**Affiliations:** 1 Nelson Institute, SAGE (Sustainability and the Global Environment), University of Wisconsin–Madison, Madison, Wisconsin, USA; 2 Department of Population Health Sciences, School of Medicine and Public Health, University of Wisconsin–Madison, Madison, Wisconsin, USA; 3 Now: Public Policy Center, University of Iowa, Iowa City, Iowa, USA; 4 Department of Atmospheric and Oceanic Sciences, University of Wisconsin–Madison, Madison, Wisconsin, USA; 5 School of City and Regional Planning, Georgia Institute of Technology, Atlanta, Georgia, USA; 6 Department of Urban and Regional Planning, University of Wisconsin–Madison, Madison, Wisconsin, USA; 7 Wisconsin Department of Natural Resources, Madison, Wisconsin, USA; 8 Global Health Institute, University of Wisconsin–Madison, Madison, Wisconsin, USA

**Keywords:** air pollution, BenMAP, bicycling, built environment, climate change, ozone, particulate matter, physical activity, urban design, vehicle emissions

## Abstract

Background: Automobile exhaust contains precursors to ozone and fine particulate matter (PM ≤ 2.5 µm in aerodynamic diameter; PM_2.5_), posing health risks. Dependency on car commuting also reduces physical fitness opportunities.

Objective: In this study we sought to quantify benefits from reducing automobile usage for short urban and suburban trips.

Methods: We simulated census-tract level changes in hourly pollutant concentrations from the elimination of automobile round trips ≤ 8 km in 11 metropolitan areas in the upper midwestern United States using the Community Multiscale Air Quality (CMAQ) model. Next, we estimated annual changes in health outcomes and monetary costs expected from pollution changes using the U.S. Environmental Protection Agency Benefits Mapping Analysis Program (BenMAP). In addition, we used the World Health Organization Health Economic Assessment Tool (HEAT) to calculate benefits of increased physical activity if 50% of short trips were made by bicycle.

Results: We estimate that, by eliminating these short automobile trips, annual average urban PM_2.5_ would decline by 0.1 µg/m^3^ and that summer ozone (O_3_) would increase slightly in cities but decline regionally, resulting in net health bene-fits of $4.94 billion/year [95% confidence interval (CI): $0.2 billion, $13.5 billion), with 25% of PM_2.5_ and most O_3_ bene-fits to populations outside metropolitan areas. Across the study region of approximately 31.3 million people and 37,000 total square miles, mortality would decline by approximately 1,295 deaths/year (95% CI: 912, 1,636) because of improved air quality and increased exercise. Making 50% of short trips by bicycle would yield savings of approximately $3.8 billion/year from avoided mortality and reduced health care costs (95% CI: $2.7 billion, $5.0 billion]. We estimate that the combined benefits of improved air quality and physical fitness would exceed $8 billion/year.

Conclusion: Our findings suggest that significant health and economic benefits are possible if bicycling replaces short car trips. Less dependence on automobiles in urban areas would also improve health in downwind rural settings.

The current fossil fuel–based transportation system of the United States negatively impacts human health by increasing air pollution and automobile accidents and by decreasing physical activity. Here, we consider how replacing short automobile trips with bicycle transport might yield health benefits through improved air quality and physical fitness, with a focus on the upper midwestern United States as our study region.

Both ozone (O_3_) and fine particular matter ≤ 2.5 µm in aerodynamic diameter (PM_2.5_) in the ambient air exacerbate bronchitis and asthma and may contribute to cardio-vascular mortality (Brunekreef and Holgate 2002). Asthma affects 8.2% of U.S. citizens, and an estimated 10 million adults have diagnosed chronic obstructive pulmonary disease (COPD) (Centers for Disease Control and Prevention 2009). In addition, recent estimates attribute 63,000–88,000 pre-mature deaths per year due to PM_2.5_ [U.S. Environmental Protection Agency (EPA) 2010c]. In the United States, on-road vehicles are responsible for about 26% of volatile organic compounds (VOCs) and 35% of nitrogen oxide (NO_x_) emissions (U.S. EPA 2005c, 2005d). NO_x_ and VOCs combine to form O_3_ and contribute to nitrate and secondary organic aerosols, important components of PM_2.5_. Nearly 240 U.S. counties, with > 118 million total residents, exceeded U.S. EPA O_3_ standards in 2011, and > 200 counties (> 88 million total residents) failed to meet PM_2.5_ standards, in part because of pollution from short car trips (U.S. EPA 2011a, 2011b).

Transport-related inactivity, that is, the use of motorized transport rather than walking and bicycling, has been linked to increased mortality and decreases in healthy life years, with the greatest impacts on chronic diseases including heart disease, stroke, colon cancer, diabetes mellitus type 2, obesity, breast cancer, and osteoporosis [World Health Organization (WHO) 2002]. Carlson et al. (2009) estimated that 32.4% of the U.S. population is fully inactive (no moderate-intensity or vigorous-intensity physical activity lasting at least 10 min at a time), while only 33.5% is physically active, defined as 30 min/day with moderate-intensity activity, ≥ 5 days/week. In a recent Dutch study, Johan de Hartog et al. (2010) concluded that shifting from short car trips to bicycle trips would reduce all-cause mortality, with estimated reductions in mortality due to increased physical activity that were nine times greater than estimated increases in mortality due to increased pollution inhalation and traffic-related fatality estimates in the Netherlands.

In the United States, 28% of all car trips are ≤ 1.6 km (1 mi), which is the distance that a typical European would walk (European Commission 2001; Pucher and Dijkstra 2003). Another 41% of all trips are ≤ 3.2 km (2 mi), a distance that many Europeans would be as likely to bicycle as to walk (European Commission 2001; Pucher and Dijkstra 2003). If we use European travel behavior as a point of comparison for walking and bicycling activity for the United States, these data suggest that many car trips in the United States could be avoided.

Amplifying the potential benefits of increased bicycle use is the nonlinear relation-ship of vehicle emissions to travel time. A large fraction of emissions (25% of VOC and 19% of primary PM_2.5_) are emitted in just the first few minutes of automobile operation, often known as “cold start,” before pollution-control devices operate [Federal Highway Administration (FHWA) 2006]. Because emissions control systems reach operating temperature only after several miles of travel and typically cool below operating range in < 1 hr (Singer et al. 1999), reducing the number of short trips could disproportionately curtail pollutant emissions from on-road vehicles.

In the present study, we quantified the potential health and monetary bene-fits of replacing short (≤ 4 km one way) car trips with travel by bicycle (50% of trips) in the 11 largest mid-western metropolitan statistical areas (MSAs): Chicago, Illinois; Cincinnati, Cleveland, Columbus, and Dayton, Ohio; Detroit and Grand Rapids, Michigan; Indianapolis, Indiana; Madison and Milwaukee, Wisconsin; and Minneapolis/St. Paul, Minnesota. This study builds on the Projected Land Use and Transportation (PLUTO) modeling framework developed by Stone et al. (2007). We estimated changes in regional emissions and air quality, as well as resulting health benefits, across the upper mid-western states [see Supplemental Material, Figure 1 (http://dx.doi.org/10.1289/ehp.1103440) for a map of the area]. In addition, we estimated the benefits of increased physical activity using the Health Economic Assessment Tool (HEAT) for cycling developed by the WHO (Rutter et al. 2007).

**Figure 1:**
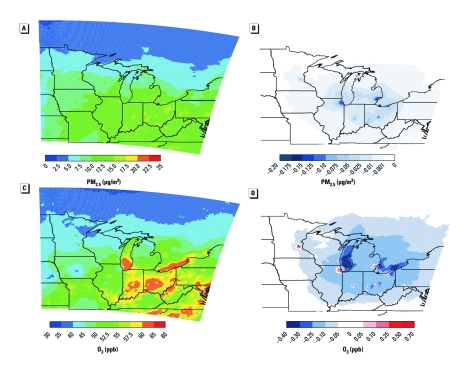
Results of air quality analysis for PM_2.5_ (*A,B*) and O_3_ (*C,D*) by location. (*A*) 2002 annual average PM_2.5_ concentration (µg/m^3^). (*B*) Estimated reduction in 2002 annual average PM_2.5_ concentration (µg/m^3^) due to changes in urban and suburban mobile emissions. (*C*) 2002 average daily 8-hr maximum O_3_ concentration (ppb) for the O_3_ season (1 May–30 September). (*D*) Estimated change in 2002 average daily 8-hr maximum O_3_ concentration (ppb) for the O_3_ season due to changes in urban and suburban mobile emissions. Data were generated in BenMAP 4.0 and mapped in ArcGIS 10 (ESRI, St. Paul, MN).

## Methods

We estimated that eliminating short car trips (≤ 8 km round trip) in urban areas of Illinois, Indiana, Michigan, Minnesota, Ohio, and Wisconsin would reduce residential vehicle use by 20%. This estimate is based on a census-tract level travel and mobile emissions inventory by Stone et al. (2007), who combined 1995 Nationwide Personal Transportation Survey (NPTS) responses (FHWA 1997), demo-graphic modeling of household vehicle travel, and the U.S. EPA MOBILE6 emissions factor model (U.S. EPA 2004b). From that contemporary emissions inventory, we estimated current emissions levels if all round trips of ≤ 8 km in urban and suburban census tracts were made using alternate modes of transportation. To inform the potential impact of a range of realistic policies and choices, we used these estimated reductions to quantify the maxi-mum potential response to a change in travel behavior. Although arbitrary, this assumed reduction in short auto trips would be consistent with the use of active (cycling or walking) transportation in European cities similar in density and population to the MSAs considered here. These values represent theoretical upper bounds on short-trip transportation choices under current travel patterns and population density. We assume that no change occurs in rural travel because distances between residential and commercial areas are typically too great for bicycling or walking and because rural populations are too low to support mass transportation.

Specifically, we compared transportation modes used in the study-area cities, with populations ranging from 837 persons/km^2^ in Grand Rapids to 4,884 persons/km^2^ in Chicago (average 2,051 persons/km^2^), to five European cities with similar population densities (range 901–5,971, average 2,910 persons/km^2^) [see Supplemental Material, Table 1 (http://dx.doi.org/10.1289/ehp.1103440)]. Although public transportation use was similar, only 39% of trips were made by automobile in the European cities, compared with 80% of trips in the Great Lakes region. Although the configurations and historical growth patterns of the European cities differ from their American counter-parts, the fact that half of all trips used active transportation suggests that active transport for 50% of short trips is feasible for similar travel distances in mid-sized American cities of similar density and that greater active transportation need not be limited to areas of highest density.

**Table 1:** Estimated PM_2.5_ reductions, reductions in health impacts, and valuation of reduced PM_2.5_ exposure.

City/data	Mean annual PM_2.5_ reduction*a *	Mortality	Asthma	Chronic bronchitis	Respiratory problems*b *	Cardiovascular problems*c *	Work-loss days*d *	Total savings
Chicago																
Incidence		0.05 (0.02, 0.15)		162 (63, 260)		802 (91, 2,301)		29 (5, 53)		36,690 (30,233, 43,145)		253 (99, 407)		5,923 (5,161, 6,685)		
Savings				1,273 (176, 3,361)		0.04 (0.01, 0.13)		13 (1, 44)		2.47 (1.44, 3.61)		16.7 (5, 40.9)		1 (0.96, 1.2)		1,305 (184, 3,449)
Cincinnati																
Incidence		0.03 (0.01, 0.10)		26 (10, 42)		100 (11, 288)		4 (0.7, 7)		4,751 (3,918, 5,583)		34 (13, 54)		763 (665, 861)		
Savings				212 (29, 549)		0.005 (0.001, 0.02)		1.6 (0.14, 6)		0.32 (0.18, 0.46)		2.27 (0.68, 5.6)		0.13 (0.11, 0.15)		212 (30, 561)
Cleveland																
Incidence		0.05 (0.02, 0.16)		53 (21, 85)		184 (21, 527)		7 (1, 13)		8,804 (7,264, 10,345)		74 (28.5, 119)		1,405 (1,224, 1,586)		
Savings				418 (58, 1,105)		0.01 (0.001, 0.03)		3 (0.3, 11)		0.60 (0.35, 0.88)		4.86 (1.4, 11.9)		0.23 (0.2, 0.27)		427 (60, 1,129)
Columbus																
Incidence		0.04 (0.02, 0.14)		27 (11, 43)		124 (14, 355)		4 (1, 8)		5,854 (4,829, 6,879)		35 (13.5, 55.9)		951 (828, 1,073)		
Savings				212 (29, 561)		0.007 (0.001, 0.02)		1.9 (0.2, 6.7)		0.39 (0.23, 0.57)		2.37 (0.71, 5.8)		0.16 (0.14, 0.18)		217 (31, 574)
Dayton																
Incidence		0.04 (0.03, 0.10)		14 (6, 23)		47 (5, 136)		2 (0.4, 3.4)		2,278 (1,880, 2,676)		18 (7, 29)		365 (318, 412)		
Savings				112 (15, 296)		0.003 (0, 0.008)		0.8 (0.07, 2.8)		0.15 (0.09, 0.22)		1.22 (0.36, 3)		0.06 (0.05, 0.07)		114 (16, 302)
Detroit																
Incidence		0.05 (0.02, 0.16)		106 (41, 171)		462 (52, 1,325)		17 (3, 31)		21,181 (17,462, 24,899)		158 (61.5, 254)		3,395 (2,958, 3,832)		
Savings				836 (115, 2,208)		0.025 (0.003, 0.08)		7.3 (0.6, 26)		1.4 (0.83, 2.09)		10.46 (0.26, 2.2)		0.66 (0.58, 0.75)		856 (120, 2,262)
Grand Rapids																
Incidence		0.03 (0.02, 0.06)		7 (3, 11)		45 (5, 130)		2 (0.3, 2.9)		2,023 (1,667, 2,379)		13 (4.75, 20.3)		327 (285, 369)		
Savings				54 (7, 143)		0.002 (0, 0.007)		0.66 (0.06, 2.3)		0.13 (0.08, 0.19)		0.88 (0.26, 2.2)		0.054 (0.047, 0.061)		56 (8, 148)
Indianapolis																
Incidence		0.03 (0.01, 0.09)		19 (7, 30)		85 (10, 243)		3 (0.5, 5.2)		3,676 (3,024, 4,328)		24 (9.3, 38.8)		592 (516, 669)		
Savings				146 (20, 386)		0.005 (0.001, 0.01)		1.2 (0.11, 4.3)		0.25 (0.14, 0.36)		1.59 (0.48, 3.9)		0.1 (0.09, 0.12)		149 (21, 394)
Madison																
Incidence		0.02 (0.02, 0.04)		1 (0.44, 1.8)		10 (1, 28)		0.42 (0.08, 0.8)		565 (469, 661)		3 (1.2, 5)		93 (81, 105)		
Savings				8.8 (1.2, 23.3)		0.001 (0, 0.002)		0.18 (0.02, 0.6)		0.037 (0.022, 0.053)		0.23 (0.067, 0.55)		0.016 (0.014, 0.018)		9 (1, 24)
Milwaukee																
Incidence		0.04 (0.02, 0.08)		12 (5, 19)		73 (8, 210)		3 (0.5, 5)		3,407 (2,809, 4,005)		21 (7.7, 34.3)		545 (475, 616)		
Savings				93 (13, 246)		0.004 (0, 0.012)		1.18 (0.10, 4.2)		0.22 (0.13, 0.33)		1.72 (0.51, 4.23)		0.095 (0.08, 0.1)		96 (14, 254)
Twin Cities																
Incidence		0.01 (0.00, 0.06)		7 (2.7, 11)		87 (10, 248)		3 (0.65, 6)		4,379 (3,619, 5,139)		27 (10, 44)		709 (618, 800)		
Savings				54 (7, 142)		0.005 (0.001, 0.014)		1.46 (0.13, 5.2)		0.28 (0.17, 0.42)		1.95 (0.58, 4.8)		0.13 (0.11, 0.15)		57 (8, 152)
Total MSAs																
Incidence		0.01 (0.00, 0.16)		433 (169, 698)		2,018 (228, 5,790)		75 (14, 137)		93,607 (77,175, 110,037)		659 (255, 1,062)		15,067 (13,128, 17,006)		
Savings				3,484 (480, 9,199)		0.109 (0.012, 333)		32 (2.8, 112)		6.16 (3.59, 9.01)		43.4 (13, 106.3)		2.6 (2.38, 3.1)		3,570 (500.7, 9,875)
Outside MSAs total																
Incidence				92 (35, 149)		541 (60.9, 1,552)		21.64 (3, 40.2)		579 (278, 878)		200.6 (71.8, 332)		4,280 (3,729, 4,830)		
Savings				726.7 (100, 1,919)		0.0227 (0.0026, 0.069)		7.35 (0.640, 26)		1.36 (0.792, 1.99)		11.1 (3.27, 27.4)		0.489 (0.426, 0.552)		747.02 (105.1, 1,975)
Region total																
Incidence				525 (204, 806)		2,559 (289, 7,342)		97 (17, 177)		94,186 (77,453, 110,915)		860 (327, 1,394)		19,347 (16,857, 21,837)		
Savings				4,143 (571, 10,937)		0.132 (0.015, 0.402)		39.1 (3.41, 138)		7.52 (4.38, 10.99)		54.5 (16.2, 132)		3.24 (2.82, 3.65)		4,247.5 (598, 11,222)
Values for incidence represent estimated incidence per adverse health effect avoided due to a change in air pollution in the given city per year; savings are presented in millions of dollars. Values in parentheses are 95% confidence intervals, and all changes are annualized. **a**Change in PM_2.5_ (µg/m^3^) was calculated as area averaged and reported with a range of minimum and maximum grid cell values; data for PM_2.5_-related health effects estimated in this analysis (and the source of the PM concentration–response functions used to estimate the change in incidence) are from Abbey et al. (1995), Dockery et al. (1996), Ito (2003), Laden et al. (2006), Moolgavkar (2000a, 2003), Norris et al (1999), Ostro (1987), Ostro and Rothschild (1989), Ostro et al. (2001), Peters et al. (2001), Pope et al. (1991, 2002), Schwartz and Neas (2000), Sheppard (2003), and Vedal et al. (1998). **b**Respiratory problems include upper and lower respiratory symptoms, hospital admissions (respiratory), emergency room visits (respiratory), and cases of acute bronchitis. **c**Cardiovascular problems include nonfatal acute myocardial infarctions and cardiovascular hospitalizations. **d**Yearly work-loss-day incidence based on estimates from the 1996 National Health Interview Survey (Adams et al. 1999).																

We estimated changes in emissions only for on-road light-duty passenger vehicles with internal combustion engines and only for round trips ≤ 8 km. We modeled changes in primary emissions (including NO_x_, carbon monoxide, sulfur dioxide, ammonia, VOCs, elemental carbon, organic carbon, and primary fine and coarse particulate matter) from all stages of vehicle operation, as well as emissions from evaporation, brake dust, resuspended road dust, and refueling. Reducing the number of short trips further lessens the frequency of cold starts from 59.9% to 21.9% of trips in urban tracts and from 55.6% to 20.3% in suburban tracts, with corresponding reductions in VOC and NO_x_ emissions. We mapped emissions from the census-tract level to the 12 × 12 km^2^ model grid by area-weighted averaging using the U.S. EPA Sparse Matrix Operator Kernel Emissions (SMOKE) model, version 2.4 (Community Modeling and Analysis System Center 2007). Emissions from sources other than motor vehicles were from the 2001 National Emissions Inventory (U.S. EPA 2005a) and were held constant in both scenarios.

We estimated changes in ambient air PM_2.5_ and O_3_ concentrations using hourly regional chemical transport simulations with the Community Multiscale Air Quality Model (CMAQ), version 4.6 (Byun and Schere 2006), driven by meteorology from the weather research and forecasting model for the full year of 2002 (Skamarock and Klemp 2008; Skamarock et al. 2008). Simulations with CMAQ were conducted on a 12 × 12 km^2^ grid and included gas phase, aqueous, and heterogeneous chemical reactions and equilibrium aerosol thermo-dynamics. We followed the model configuration used by Spak and Holloway (2009), with boundary conditions from a 36 × 36 km^2^ simulation over continental North America.

We used the Environmental Benefits Mapping and Analysis Program (BenMAP) version 4.0.35 (U.S. EPA 2010a) to estimate health impacts due to CMAQ-simulated changes in ambient air pollution resulting from reduced car travel. Because BenMAP addresses both mobile and stationary sources (U.S. EPA 2004a, 2008), it has been used to support the creation of environ-mental regulations in several countries.

After air quality data is loaded into BenMAP, the program determines the change in ambient air pollution. BenMAP then uses concentration–response functions (CR) to calculate the relationship between the pollution and certain health effects, applying the relationship to the exposed population (Abt Associates 2010). Finally, BenMAP uses a “damage function” to estimate health costs and benefits from changes in air quality. A damage function quantifies the health benefits and economic value of reduced exposure to pollutants (Davidson et al. 2007).

BenMAP 4.0 (i.e., version 4.0.35) incorporates hourly air pollution data and county-level baseline incidence rates for the following health outcomes: overall mortality, asthma exacerbations, chronic bronchitis, hospital admissions, acute myo-cardial infarctions, acute and chronic respiratory infections, upper and lower respiratory infections, work-loss days, and school-loss days. Spatial speci-ficity in baseline incidence data varies by health outcome and location; where county-level data are not available, BenMAP distributes state estimates to the county level using age-specific rates for each health outcome within each county. For mortality estimates, BenMAP combines national-level census mortality rate projections and county-level age-specific incidence rates from 2006 with projected changes in study area populations to derive county-level mortality rate projections for 2010. For the present study, BenMAP used state-level hospitali-za-tion data to estimate county-level incidence for Minneapolis/St. Paul, Chicago, and Indianapolis; county-level incidence data for all cities in Ohio; and city hospital discharge data for Milwaukee, Madison, Detroit, and Grand Rapids. For emergency room (ER) admissions, we used midwest regional incidence data for Detroit, Grand Rapids, Chicago, and Indianapolis; state-level data for Minneapolis/St. Paul; county-level data for Ohio cities; and hospital discharge-level data for Milwaukee and Madison. For all cities, non-fatal acute myo-cardial infarction incidence rates were based on regional hospitalization data. All other health end point data were based on national figures.

BenMAP assigns monetary values to the reduction of adverse health effects based on national averages that do not reflect intra-city or inter-city variability in costs. The BenMAP analysis was conducted on the 12 × 12 km^2^ grid, using 2010 census projection allocation to the grid by the U.S. EPA. Valuation is in 2010 dollars.

We combined air quality estimates for 2002 from CMAQ with 2002 U.S. EPA monitoring using spatial scaling by Voronoi nearest neighbor averaging (e.g., Chen et al. 2004). This pairing yields air quality inputs to BenMAP including complete spatial and temporal coverage by high-resolution hourly modeling, constrained to match concentrations observed near monitors. We then used the expert-derived PM_2.5_ CR functions, valua-tion estimates, and pooling methods used for the U.S. EPA 2006 Regulatory Impact Analysis, plus O_3_ exposure–response functions for 2008 National Ambient Air Quality Standard (NAAQS) evaluations (U.S. EPA 2004a, 2008; University of North Carolina Institute for the Environment, Community Modeling and Analysis System Center 2008). Because multiple studies exist for each given health incidence, pooling techniques are often used to statistically combine the results. Using BenMAP, we ran each CR function and pooling of incidence and valuation for each health end point in a 5,000-member Monte Carlo ensemble. Sources of CR functions used in this analysis are presented in Tables 1 and 2. As standard practice, the U.S. EPA does not pool mortality studies. Thus, we used the Harvard Six Cities study (Pope et al. 2002) as BenMAP input for PM_2.5_ mortality; that study included the most representative sites. We selected the 2010 population database to use in BenMAP because the sensitivity studies we conducted indicated that choice of year has no substantial impact (1–2% difference) on incidence of health threats.

**Table 2:** Estimated O_3 _changes, changes in health impacts, and valuation of changes in O_3_ exposure.

City/data	Change in O_3_ (ppm)*a*	Acute respiratory symptoms	ER visits (respiratory)	HA (respiratory)	Mortality	School-loss days	Worker productivity	Total savings
Chicago									
Incidence		–0.09 (–0.23, 0.39)	–5,780 (–10,131, –1,431)	–3 (–12, 2.4)	–8 (–18. 0.87)	–14 (–22, –6)	–1,762 (–3,174, –351)	–13,564	
Savings			–0.365 (–0.718, 0.047)	–0.001 (–0.004, 0.001)	–0.19 (–0.41, 0.03)	–108 (–305, 38)	–0.17 (–0.3, –0.03)	–0.017	–109 (–306, 37)
Cincinnati									
Incidence		–0.13 (–0.25, 0.21)	483 (–229, 1,194)	0.08 (–1.1, 1.3)	0.53 (–0.72, 1.76)	0.78	151 (–75, 377)	1,279	
Savings			0.03 (–0.02, 0.09)	0 (0, 0)	0.011 (–0.02, 0.04)	6.2 (–20.2, 37.3)	0.014 (–0.007, 0.036)	0.002	6.2 (–20.2, 37.5)
Cleveland									
Incidence		–0.17 (–0.33, 0.70)	353 (–871, 1577)	–0.15 (–2.6, 2.2)	0.35 (–2.2, 2.8)	0.54 (–2.3, 3.4)	106 (–271, 483)	2,490	
Savings			0.022 (–0.07, 0.12)	0 (–0.001, 0.001)	0.008 (–0.05, 0.06)	4.4 (–47.4, 61)	0.01 (–0.03, 0.05)	0.003	4.412 (–47.5, 61.3)
Columbus									
Incidence		–0.2 (–0.27, 0.23)	199 (–555, 953)	–0.07 (–1.3, 1.09)	0.19 (–0.9, 1.3)	0.45 (–0.85, 1.8)	58 (–182, 297)	1,697	
Savings			0.013 (–0.04, 0.07)	0 (0, 0)	0.005 (–0.02, 0.03)	3.64 (–20, 29.7)	0.005 (–0.017, 0.028)	0.002	3.669 (–20, 29.9)
Dayton									
Incidence		–0.21 (–0.26, 0.01)	713 (362, 1,064)	0.48 (0, 1.4)	0.99 (0.26, 1.8)	2 (1.3, 2.9)	209 (100, 318	2,568	
Savings			0.045 (0.02, 0.08)	0 (0, 0.001)	0.02 (0.004, 0.038)	16 (2.5, 40.6)	0.02 (0.01, 0.03)	0.003	16.462 (2.6, 40.7)
Detroit									
Incidence		–0.17 (–0.29, 0.18)	830 (–976, 2,634)	0.46 (–1.7, 3)	0.58 (–2.3, 3.3)	1 (–2.5, 4.7)	257 (–326, 840)	1,389	
Savings			0.052 (–0.07, 0.19)	0 (–0.001, 0.001)	0.011 (–0.05, 0.07)	8.5 (–48, 68)	0.024 (–0.031, 0.08)	0.002	8.631 (–48.5, 68.7)
Grand Rapids									
Incidence		–0.23 (–0.29, 0.07)	1,124 (571, 1,677)	0.65 (0, 1.9)	1 (0.34, 1.8)	2 (1.5, 3.4)	363 (174, 552)	12,235	
Savings			0.071 (0.033, 0.12)	0 (0, 0)	0.02 (0.005, 0.035)	19.3 (3, 48)	0.035 (0.02, 0.05)	0.015	19 (3, 48)
Indianapolis									
Incidence		–0.15 (–0.22, 0.12)	532 (13, 1,051)	0.32 (–0.39, 1.3)	0.72 (–0.38, 1.8)	1 (0.23, 2.1)	169 (–9.2, 347)	2,036	
Savings			0.034 (–0.002, 0.076)	0 (0, 0)	0.017 (–0.009, 0.04)	9.4 (–8, 33)	0.016 (–0.001, 0.033)	0.003	9.476 (–8, 33)
Madison									
Incidence		–0.12 (–0.16, 0.04)	135 (33, 237)	0.03 (–0.01, 0.11)	0.11 (0.02, 0.21)	0.25 (0.12, 0.39)	41 (12, 70)	436	
Savings			0.009 (0.002, 0.017)	0 (0, 0)	0.002 (0, 0.004)	1.99 (0.28, 5.2)	0.004 (0.001, 0.007)	0.001	2.002 (0.28, 5.2)
Milwaukee									
Incidence		–0.12 (–0.20, 0.14)	134 (–292, 559)	–0.09 (–0.74, 0.45)	0 (–0.61, 0.57)	0.2 (–0.6, 1.1)	21 (–116, 158)	–60	
Savings			0.008 (–0.02, 0.04)	0 (0, 0)	0.001 (–0.01, 0.01)	1.65 (–11.5, 16.9)	0.002 (–0.01, 0.02)	0	1.66 (–11.6, 16.9)
Twin Cities									
Incidence		–0.05 (–0.11, 0.38)	–2,190 (–3,590, –790)	–0.76 (–2.5, 0.24)	–2 (–3.7, –0.3)	–4 (–6.5, –2.3)	–588 (–994, –181)	–7,881	
Savings			–0.138 (–0.25, –0.039)	0 (–0.001, 0)	–0.039 (–0.07, –0.003)	–34.7 (–88.9, 2.57)	–0.056 (–0.095, –0.0017)	–0.01	–34.9 (–89.3, 2.5)
Total MSAs									
Incidence		–0.07 (–0.33, 0.70)	–3,467 (–15,663, 8,723)	–963 (–8,281, 5,764)	–5.97 (–28, 16)	–9 (–32, 14)	–976 (–4,860, 2,909)	2,627	
Savings			–0.22 (–1.15, 0.72)	–0.00096 (–0.0083, 0.0058)	–0.134 (–0.63, 0.36)	–71.7 (–543, 380)	–0.093 (–0.46, 0.28)	0.0033	–72.14 (–545.38, 381.47)
Outside MSAs total									
Incidence			33,628 (17,087, 50,167)	17 (0, 51.5)	47 (10.9, 83)	91 (53.3, 129.6)	9,608 (4,610, 14,606)	282,669	
Savings			212 (0.995, 3.6)	0.0073 (0, 0.019)	1.01 (0.222, 1.81)	763.2 (118, 1,1893)	0.914 (0.439, 1.39)	0.353	767.6 (120, 1,900)
Region total									
Incidence			30,161 (1,423, 58,891)	14.63 (–22.4, 66.8)	41.13 (–17.2, 98.8)	82.6 (21.3, 143.3)	8,632 (–250.2, 17,515)	285,296	
Savings			1.91 (–0.14, 4.33)	0.0062 (–0.0083, 0.025)	0.876 (–0.42, 2.17)	691.5 (–425, 2,273)	0.822 (–0.0239, 1.67)	0.357	695.5 (–425, 2,281)
Values for incidence represent estimated incidence per adverse health effect avoided due to a change in air pollution in the given city per year; costs are expressed as negative and benefits as positive (millions of dollars). Values in parentheses for incidence and savings are 95% confidence intervals (in most cases rounded to nearest decimal), and all changes are annualized. **a**Change in O_3_ season average daily maximum 8-hr are calculated as mean area (range of grid cell values). Data for O_3_-related health effects (and the source of the O_3 _concentration–response functions used to estimate the change in incidence) estimated in this analysis are from Bell et al. (2004, 2005), Burnett et al. (2001), Chen et al. (2000), Crocker et al. (1981), Gilliland et al. (2001), Huang et al. (2005), Ito et al. (2005), Jaffe et al. (2003), Levy et al. (2005), Moolgavkar et al. (1997), Ostro and Rothschild (1989), Peel et al. (2005), Schwartz (1994a, 1994b,1995, 2005), and Wilson et al. (2005).									

To address the potential health and economic co-benefits that would result if half of all short trips were made by bicycle, we used HEAT. This model uses relative risk data (Anderson et al. 2000) to estimate cost savings from reduced all-cause mortality. Controlling for socio-economic variables (e.g., age, sex, smoking) and leisure time activity, HEAT calculates risk reduction for days spent cycling based on estimates of total number of days cycled, distance, and average speed (Rutter et al. 2007).

We used HEAT analysis to estimate the monetized health benefits associated with the conversion of one-half of short trips (< 8 km round trip) by car to be made by bicycle. This represents 10% of vehicle miles traveled (VMT) for the region. We used the U.S. EPA value of a statistical life ($7.4 million) (U.S. EPA 2010b) and the annual percentage of all-cause working-age mortality [0.00390; 95% confidence interval (CI): 0.00277, 0.00503] (Wilkinson and Pickett 2008). We assumed an average of 124 days of cycling per year, HEAT’s default value (Rutter et al. 2007), which is representative of the climate of the upper midwest, where bicycle commuting is most common from April through October. We also assumed that only 50% of these trips would be under-taken by people who do not currently cycle, thus excluding the small percentage of the population already benefitting from cycling, as well as elderly individuals or those physically unable to bicycle. We used the NPTS average commute distance for each MSA (from 3.34 to 3.98 km) with an average speed for commuter cyclists of 14 kph. Finally, we used the HEAT-recommended default percentage (90%) of cyclists completing a round trip each day.

## Results

Simulations yielded unique hourly estimates of surface-level PM_2.5_ throughout the year (Figure 1A) and O_3_ during the warm season (1 May 30–September) (Figure 1C) on a 12 × 12 km^2^ grid for 2002. The CMAQ simulations described here captured spatial and temporal variability in PM_2.5_ [see Supplemental Material, Table 2 (http://dx.doi.org/10.1289/ehp.1103440)] and O_3_ (see Supplemental Material, Table 3) when compared with U.S. EPA monitoring data throughout the region, with performance for PM_2.5_ and O_3_ both exceeding community and U.S. EPA expectations for chemical transport modeling in policy and research applications.

**Table 3:** Results of HEAT analysis assuming that 50% of short trips are completed by bicycle.

MSA	No. of trips by bicycle (millions)	No. of trips/day (millions)	Average distance (km)	Maximum annual benefit (millions of $)*a*	Savings/individual cyclist/year ($)	Savings per trip ($)	Mean annual benefit (millions of $)	No. of people to benefit	Lives saved
Suburban																		
Chicago		280.77		0.77		3.65		972		2,298		10.19		724		211,566		131
Cincinnati		53.15		0.15		3.55		179		2,235		9.91		133		40,047		24
Cleveland		93.02		0.26		3.55		313		2,235		9.91		233		70,083		42
Columbus		51.19		0.14		3.55		172		2,235		9.91		128		38,567		23
Dayton		33.09		0.09		3.55		111		2,235		9.91		83		24,935		15
Detroit		197.11		0.54		3.55		664		2,235		9.91		495		148,506		90
Grand Rapids		35.14		0.10		3.98		133		2,506		11.11		99		26,474		18
Indianapolis		57.46		0.16		3.98		217		2,506		11.11		162		43,294		29
Madison		25.23		0.07		3.64		87		2,291		10.16		65		19,010		12
Milwaukee		50.85		0.14		3.64		176		2,291		10.16		131		38,314		24
Twin Cities		109.30		0.30		3.58		371		2,253		10		277		82,346		50
Total suburban		986.31		2.70		3.66		3,396		2,302		10.21		2,530		743,142		459
Urban																		
Chicago		197.16		0.54		3.43		652		2,160		9.58		4,859		151,022		88
Cincinnati		29.03		0.08		3.34		92		2,103		9.33		685		21,870		12
Cleveland		49.56		0.14		3.34		157		2,103		9.33		1,170		37,338		21
Columbus		33.45		0.09		3.34		106		2,103		9.33		790		25,205		14
Dayton		10.08		0.03		3.34		34		2,103		9.33		252		7,597		5
Detroit		62.70		0.17		3.54		211		2,228		9.89		1,569		47,238		28
Grand Rapids		10.64		0.03		3.54		36		2,228		9.89		266		8,013		5
Indianapolis		21.21		0.06		4.47		90		2,815		12.48		670		15,982		12
Madison		NA		NA		3.79		NA		NA		NA		NA		NA		NA
Milwaukee		30.80		0.08		3.79		111		2,386		10.58		83		23,208		15
Twin Cities		58.97		0.16		3.5		196		2,203		9.77		146		44,430		26
Total urban		503.60		1.38		3.58		1,684		2,039		9.05		1,255		381,902		228
Grand total		1489.92		4.08		3.62		5,080		2,171		9.63		3,784		112,5045		687
NA, not applicable. **a**The maximum annual benefit is the total value of reduced mortality based on the level of cycling entered by the user.																		

We estimated that substitution of non-emitting modes for short trips would achieve average annual reductions in the 24-hr average PM_2.5_ concentrations considered in U.S. PM_2.5_ regulations (Figure 1B). Regional O_3_ would also be reduced throughout the May–September summer season (calculated based on daily maximum 8-hr and 1-hr averages, consistent with U.S. O_3_ regulations) but daytime O_3_ would increase in the largest cities because of VOC-limited O_3_ production conditions in urban environments (Figure 1D). Effects of transportation on O_3_ concentrations within the MSAs are complex because of the non-linear inter-play of emissions and meteorology in atmospheric chemistry and transport, whereby local ambient O_3_ concentrations often increase in response to reductions in NO_x_ and/or VOC emissions (Sillman 1995). In our emissions inventory, motor vehicles were responsible for most of the NO_x_ (70–98%) and VOC (40–95%) emissions in the MSAs, with the highest percentages of emissions from motor vehicles in the most urbanized areas. Although Figure 1B and D show long-term averages (annual for PM_2.5_ and summer for O_3_), we used hourly values from CMAQ to estimate the potential health benefits of increased active transport.

*Fine particulates (PM2.5). *We observed changes in PM_2.5_ and O_3_, associated health outcomes, and monetary savings for each MSA and for the combined total of all grid cells outside the 11 MSAs (Tables 1 and 2). We estimated that eliminating short car trips would reduce annual average PM_2.5_ across the study region by 0.08–0.15 µg/m^3^ (1.0–2.0%) in most MSA urban centers. In the upwind MSAs of Madison and Minneapolis/St. Paul, which would see little bene-fit from PM_2.5_ reductions in other cities, we estimated that PM_2.5_ would be reduced by 0.05 µg/m^3^ (Figure 1B). Nearly all of the estimated reduction in PM_2.5_ would be due to decreases in secondary aerosols, especially nitrate formed from NO_x_ and secondary organic aerosols from VOCs. Primary particle emissions from motor vehicles are negligible, so the reduced VMT scenario would not significantly affect this smaller fraction of PM_2.5_ mass. Reductions in PM_2.5_ in urban areas and downwind would be greatest during high-pollution episodes exceeding the 24-hr average PM_2.5_ NAAQS. In urban grid cells, the average estimated reduction during NAAQS exceedances was 0.20 µg/m^3^, equivalent to the maximum change in annual average PM_2.5_ in Chicago [see Supplemental Table 4 (http://dx.doi.org/10.1289/ehp.1103440)]. In addition, we estimated that the reduction in short auto trips would result in one fewer exceedance per year in a typical urban grid cell and a 5–25% reduction in the number of annual exceedances.

**Figure 2:**
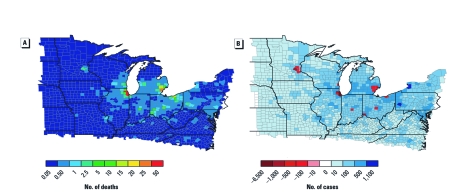
Examples of altered incidence of negative health outcomes by county. (*A*) Annual reduction in premature mortalities due to reduced PM_2.5_; units reflect the reduction in number of deaths per year. (*B*) Annual reduction in cases of acute respiratory symptoms due to changes in O_3_; units reflect the number of cases of acute respiratory symptoms per year. Data were generated in BenMAP 4.0 and mapped in ArcGIS 10.

Our results indicate that adverse health outcomes related to PM_2.5_ would be reduced in all MSAs (Table 1). Reductions in PM_2.5_-related mortality across the midwest are shown in Figure 2A, with the total impact across the 37,000-mi^2^ region being 525 fewer deaths. We estimated that asthma exacerba-tions would decrease annually by > 2,500 cases. In addition, there would be approximately 100 fewer COPD cases, whereas net respiratory symptoms, hospital admissions, and ER visits would decrease by 94,186 cases annually. Regarding cardio-vascular disease, there would be approxi-mately 860 fewer cases of non-fatal acute myocardial infarction and hospital admissions. Savings from reduced annual mortality would reach almost $4.14 billion. Savings of > $7.5 million would result from fewer respiratory cases, hospital admissions, and ER visits, whereas a reduction in COPD would save > $39 million per year; reductions in non-fatal acute myo-cardial infarctions and cardio-vascular hospitalizations would save > $54 million. We estimate that total savings from reducing adverse health effects due to PM_2.5 _would be about $4.25 billion/year (95% CI: $598 million–$11.2 billion). Projections suggest that PM_2.5_ exposure would also be reduced in populations outside MSAs and that resulting reductions in adverse health effects would account for roughly 25% of the total benefit.

*Ozone.* Estimated effects of eliminating short car trips on O_3_ pollution vary in relation to the size and density of urban areas. For large urban areas, estimated daily 8-hr maximum, 1-hr maximum, and daily average O_3_ concentrations during the May–September O_3_ season generally increased in city centers, whereas concentrations decreased in suburbs, some smaller urban areas, and in areas downwind of the MSAs (Figure 1D). Simulated changes in transportation and reductions in cold-start frequency would decrease total NO_x_ emissions by 5–12% and total VOC emissions by 10–25%.

Although we estimate that NO_x_ and VOCs would both be reduced, the response to NO_x_ reductions would be more pronounced, resulting in increased O_3_ in urban cores, consistent with previous studies in the region (Sillman 1995). Changes in estimated O_3_ concentrations were greater during the warmest months (July–August) when concentrations are highest, with increases and decreases of up to 2 ppb. We estimate that daily 8-hr maximum O_3_ would increase on a population-weighted basis (Table 2) but that area-averaged O_3_ levels would decrease in every MSA. BenMAP analysis indicated net regional savings from declines in mortality, school-loss days, hospitalizations, ER visits, and acute respiratory symptoms, but some increases in costs in cities such as Chicago, Cleveland, Columbus, Milwaukee, and Minneapolis/St. Paul due to changes in O_3_ levels. Costs resulting from O_3_ increases due to reduced VMT were statistically significant for only Chicago and Minneapolis/St. Paul, but estimated savings from PM_2.5_ reductions were greater than increased costs due to O_3_ in all cities.

We estimated that areas outside the MSAs would experience net benefits for all O_3_-related health outcomes. For nine of the cities (excluding Chicago and Minneapolis/St. Paul), we estimated a potential reduction of approximately 30,000 cases in acute respiratory symptoms associated with the potential changes in O_3_ (resulting in savings of almost $1.9 million) and 8,632 fewer school-loss days (savings of almost $822,000). This distinct reduction in acute respiratory symptoms to areas outside the MSAs is shown in Figure 2B.

Estimated changes in health outcomes due to changes in O_3_ are less correlated with MSA density or size than estimated changes due to reduced PM_2.5_, particularly for outcomes related to daily peak values, such as acute respiratory symptoms. Instead, estimated changes in O_3_-related health impacts were often more pronounced in smaller MSAs such as Dayton and Grand Rapids, reflecting differences in total VOC:NO_x_ ratios and the degree to which reductions in local motor vehicle emissions would alter them. Thus, estimated effects of eliminating short car trips on population O_3_ exposures are highly sensitive to urban size, density, and travel patterns.

*Benefit from physical activity.* Based on WHO HEAT analysis, we estimated that completing 50% of short trips by bicycle would result in average annual savings of > $2.5 billion for short suburban bicycle trips and nearly $1.25 billion for short urban trips (Table 3), for a total of approximately $3.8 billion in bene-fits across an estimated population of 2 million people and a reduction in pre-mature mortality of almost 700 deaths/year.

## Discussion

In the study region with a population of 31.3 million, we estimated that eliminating short car trips and completing 50% of them by bicycle would result in mortality declines of approximately 1,295 deaths/year (95% CI: 912, 1,636), including 608 fewer deaths due to improved air quality and 687 fewer deaths due to increased physical activity. Changes in PM_2.5_ and O_3_ would result in net health bene-fits of $4.94 billion/year (95% CI: $0.2 billion, $13.5 billion). Completing 50% of short trips by bicycle would yield $3.8 billion/year in savings (95% CI: $2.7, $5.0 billion), about $1.5 billion less in savings than from reductions in air pollution. We estimate that the combined benefit from improved air quality and physical fitness for the region would exceed $8.7 billion/year, which is equivalent to about 2.5% of the total cost of health care for the five midwestern states in the present study in 2004 (Kaiser Family Foundation 2004).

Of course, an added benefit of removing 20% of VMT from the region is also reduced emissions of greenhouse gases that cause global climate change. The annual reduction would be > 1.8 teragrams carbon dioxide (CO_2_) (3.9 billion pounds), using the fleet average passenger car fuel economy of 22.1 mi/gal, with 1 gal gasoline producing 0.882 lb CO_2_ (U.S. EPA 2005b).

Few studies have addressed how changes in behavior can affect air quality (Frank and Engelke 2005; Frank et al. 2000), and none have quantified the potential benefits of travel behavior change for pollution control. Comparison with prior BenMAP cost–benefit regulatory analyses suggests that health bene-fits from reduced air pollution through behavioral changes in personal transportation would be comparable with effects of such top-down meas-ures as the Clean Air Interstate Rule and the Nonroad Diesel Rule, both air quality regulations having potential for substantial impacts on human health (Hubbell et al. 2009). The magnitude of regional impacts from urban travel mode substitution would be comparable with the annualized benefit of reducing O_3_ nationwide to full compliance with the current 75 ppb NAAQS (U.S. EPA 2008).

Compliance with federal air quality standards through conventional measures such as emissions controls entails direct costs to govern-ments and private industry. In contrast, changing personal travel behavior distributes costs and bene-fits—both financial and otherwise—in a more complex manner, including potentially large personal savings for individuals given the high cost of vehicle owner-ship and operation. However, in addition to public outreach, education, and incentive programs, drastic decreases in residential VMT would require infrastructure investments to support pedestrian and bicycle traffic, as well as increased public transit. For example, cities would need to designate bicycle lanes on streets, add bicycle lanes or mixed-use nonmotorized paths, and provide additional signage, physical barriers, bicycle traffic signals, and bicycle parking. Infrastructure costs for converting existing roadways to bicycle lanes in the United States range from $2,500 to $50,000/block, depending on the infrastructure needs. In 2010 Portland, Oregon, converted 10 blocks of high-traffic streets to include two-way bike lanes at a cost of $10,000/block, reducing motor vehicle traffic by one lane. In 2011, Chicago added protected bicycle lanes with flexible marker posts and a parking lane for automobiles along four blocks, including a bridge, at a cost of $140,000 (City of Chicago 2011). Increasing this cost estimate to $100,000/block, double the U.S. average cost per mile for bike lane conversion and addition, the $2 billion in health cost savings in the MSA of Chicago alone could retrofit 20,000 blocks (2,500 mi or 4,020 km) with bike lanes. The greater Chicago metropolitan area has > 23,500 mi of urban roads, not including interstate or freeways (Illinois Department of Transportation 2009), so the health care savings could cover the costs of adding bike lanes to every road in 1–10 years.

Although U.S. pedestrians and cyclists may be at higher risk of mortality than their Dutch counterparts (Pucher and Dijkstra 2003), the Dutch results provide a model for safer walking and cycling. Seven of the cities studied here—Chicago, Columbus, Dayton, Indianapolis, Madison, Milwaukee, and Minneapolis/St. Paul—have earned bicycle-friendly rankings from the League of American Bicyclists because they actively support bicycling by providing safe accommodation for cycling and encouraging people to bike (League of American Bicyclists 2010). Thus, some U.S. communities may be more likely than others to exhibit charac-teristics of Dutch cities that make bicycling feasible. There is already an observed trend of increasing bicycle share across all of the 11 midwestern MSAs, one that is consistent and very large (U.S. Census Bureau 2009). Moreover, there is evidence that U.S. cities with enhanced levels of active transport experience health benefits. Pucher et al. (2010) found that cities with the highest rates of commuting by bicycle or on foot have obesity and diabetes rates 20% and 23% lower, respectively, than cities with the lowest rates of active commuting.

*Strengths, limitations, and uncertainties.* Our research, for the first time, has joined models of health effects (BenMAP), census-based vehicle use and emissions (PLUTO), and regional air pollution (CMAQ) to link highly localized changes in travel behavior to regional health outcomes. We also used the newest version of U.S. EPA BenMAP (4.0), which includes baseline incidence rates at the county (versus the regional) level, thus providing greater local specificity than previously possible.

Our results may be a conservative estimate of pollution reductions. We did not evaluate changes in exposure for people who live or work near highways, nor did we assess health effects from decreases in other pollutants (e.g., carbon monoxide, sulfur dioxide) or the synergistic effects of combined changes in O_3_ and PM_2.5_. We would expect the reduction in the number of automobiles on the road at any given time to change average speeds and resultant emissions, with variable effects on arterial and local roadways. Comprehensive analysis would require travel-demand modeling (e.g., Bowman and Ben-Akiva 2001) incorporating traveler decision making, spatially specific changes in roadway and transit networks, demographic information, and employment data to calculate those differences in vehicle activity. Finally, health impacts from changes in long-range transport of O_3_ and PM_2.5_ to states downwind of the modeling domain and to neighboring Canadian regions were not analyzed.

Our health benefits analysis also may be conservative because, following current U.S. EPA practice, we used total PM_2.5_ mass and did not differentiate between aerosol species. Recent epidemiological studies suggest that traffic-related emissions may contain more hazardous particulate chemical components. Gent et al. (2009) found more frequent asthma symptoms and inhaler use in children after exposure to PM_2.5_ emissions attributable to motor vehicles compared with emissions from other sources. Bell et al. (2009) found differing associations between cardio-vascular and respiratory hospitalization across various chemical species of PM_2.5_. Particles comprising vanadium, nickel, and elemental carbon showed the strongest associations (vanadium and nickel come primarily from transportation emissions). However, because these epidemiological studies included high diesel truck traffic and its specific emissions profile, these results have slightly less bearing on our analysis of decreases in light-duty automobile emissions.

Our estimates for physical fitness bene-fits stemming from bicycling 50% of short car trips (≤ 8 km) may under-estimate the full benefits of removing these car trips. Not included are the remaining trips that presumably would be achieved by some form of mass transportation or direct walking for very short trips. According to the 2001 National Household Travel Survey, Americans who use mass transit spend a median of 19 min daily walking to and from transit (Besser and Dannenberg 2005). Accounting for fitness benefits from this mode of active transport would involve complex geo-spatial modeling. Future analyses should consider geographic information system (GIS) technologies in conjunction with energy expenditure measure-ment tools, such as accelerometers or biometric monitors, to more accurately assess the speed, distance, intensity, and terrain of the cyclist (Bonnel et al. 2009). Finally, for urban planning purposes, assumptions for determining levels of benefits for new bicyclists will stem from city-specific estimates of current bicycling levels and city-wide demographics. We used current European bicycling levels to guide our maxi-mum benefit level potentially achievable.

In our study we used chemical transport modeling simulations and empirical CR functions, an experimental framework that adds incremental uncertainty at each step: in the emissions inventory, modeled meteorology, and processes included in the chemical transport model. In addition, the ability of the model to reproduce observed ambient surface-level O_3_ and PM_2.5_ and their respective sensitivities to emissions changes adds uncertainty. We used the same suite of response functions and pooling chosen by the U.S. EPA for air pollution rule making; however, the empirical epidemiological CR functions of BenMAP and the choice of valuation estimates are an additional source of uncertainty. The valuation estimates are a function of BenMAP, based on the configuration used by the U.S. EPA. Sensitivity analysis by the California Air Resources Board confirmed that the mean and distribution of premature mortalities from long-term exposure to PM_2.5_ are not sensitive to the random-effects pooling of CR functions (Tran et al. 2009). We found few outliers among the individual CR calculations that contribute to the reported pooled values. Although we chose to simulate a year (2002) that is representative of the regional climate of the past decade, the magnitude of bene-fits achieved in any given year depends on inter-annual variability in meteorology and the resultant ambient air quality.

## Conclusion

Our study demonstrates that reduced car travel and enhanced bicycle commuting in urban areas can improve health outcomes within urban, suburban, and even in downwind rural areas. Our results demonstrate that reduced car travel can benefit air quality, human health, and the economy.

## Correction

In the manuscript originally published online, the weight of the annual reduction of CO_2_ was noted in the “Discussion” as “3.9 trillion pounds” instead of “3.9 billion pounds,” and information on health bene-fits accruing outside the MSA regions was inadvertently omitted. Information for outside the MSA regions and for subsequent savings for the entire region is now included in Tables 1 and 2, and all values have been corrected here.

## Supplemental Material

(217 KB) PDF
