# Supplementary material for: Air Quality and Exercise-Related Health Benefits from Reduced Car Travel in the Midwestern United States
Source: Environ Health Perspect. 2011 Nov 2;120(1):68–76. doi: 10.1289/ehp.1103440 (PMC3261937; doi:10.1289/ehp.1103440)
Supplement: (217 KB) PDF [file ehp.1103440.s001.pdf]

Supplemental Material

Air Quality and Exercise-Related Health Benefits from Reduced Car Travel in the Midwestern United States

Authors:
Maggie L. Grabow,[1,2]
Scott N. Spak,[1,3,4]
Tracey Holloway,[1,4]
Brian Stone Jr.,[6]
Adam C. Mednick,[1,5,7]
Jonathan A. Patz[1,2,8]

Table of Contents

Supplemental Material Table 1 ……….Transportation Shares of 11 Cities in the Midwest………....Page 2

Supplemental Material Table 2………..Performance of Air Pollution Models for $PM_{2.5}$…................Page 3

Supplemental Material Table 3………..Performance of Air Pollution Models for O_3……….............Page 3

Supplemental Material Table 4………..$PM_{2.5}$ 24-hour exceedances………………………………....Page 4

Supplemental Material Figure 1……….Regional Population Density of Study Area…….............Page 5

References…………………………………………………………………………………….Page 6

Supplemental Material, Table 1. 2008 estimated population, density, transportation shares, and increase in bicycle commuting for the 11 MSAs (U.S. Census 2008) and equivalent contemporary estimates for 5 European cities (ECF 1998).

			Transportation Share					
City	Population	Population Density[a]	MSA Population	Walk	Bike	Public Transit	Car	% Inc.[b]
Chicago	2,741,455	4,884	9,569,624	6%	1.0%	27%	61%	109%
Cincinnati	294,771	1,650	2,155,137	4%	0.5%	11%	80%	137%
Cleveland	408,101	2,381	2,088,291	5%	0.7%	12%	79%	203%
Columbus	740,086	1,373	1,773,120	3%	0.9%	3%	89%	164%
Dayton	166,179	1,101	836,544	6%	0.4%	7%	82%	-
Detroit	777,493	2,463	4,425,110	2%	0.3%	9%	84%	98%
Grand Rapids	193,396	1,711	776,833	3%	1.5%	4%	86%	-
Indianapolis	798,594	837	1,715,459	2%	0.3%	2%	92%	41%
Madison	228,775	1,170	561,505	8%	3.9%	8%	75%	-
Milwaukee	581,099	2,400	1,549,308	4%	1.1%	9%	83%	231%
Minn./St. Paul	360,914	2,595	3,229,878	6%	4.3%	14%	70%	126%
Average	662,806	2,051	2,607,346	5%	1.3%	10%	80%	139%
European Cities								
Copenhagen[c]	526,918	5,971	-	12%	20%	18%	50%	
Groningen[d]	185,000	2,324	-	17%	48%	5%	30%	
Munster[e]	272,951	901	-	21%	34%	7%	38%	
Salzburg[f]	150,269	2,288	-	23%	19%	13%	45%	
Utrecht[d]	300,000	3,068	-	23%	32%	11%	34%	
Average	287,028	2,910	-	19%	31%	11%	39%	

[a] persons/km^2
[b] % Increase in Bike Commute, 2000-2008
[c] Denmark
[d] The Netherlands
[e] Germany
[f] Austria

Supplemental Materials, Table 2 and Table 3

The two tables below relate to the accuracy of our air pollution models. The model performs well for particulate speciation in our region (Spak and Holloway 2009), exceeding community performance goals throughout the year for all fine particle species except organic mass. The CMAQ simulations described here captured spatial and temporal variability of $PM_{2.5}$ and O_3 in routine EPA monitoring throughout the region, with performance for $PM_{2.5}$ and ground-level O_3 both exceeding community and EPA expectations for chemical transport modeling in policy and research applications. Based on community modeling standards, comparisons between predicted-observed pairings for 24-hour $PM_{2.5}$ samples at rural –Interagency Monitoring of Protected Visual Environment (IMPROVE) – and urban EPA Speciation Trends Network (STN) sites across the study domain (Table 6) are good to excellent in all but November-February and January-February (STN) (Morris et al 2005), meet criteria expectations at both networks in every month, and exceed performance goals (Boylan and Russell, 2006) in all but those winter months. Predicted-observed pairings for summertime hourly O_3 observations at EPA AQS monitors in the region (Table 7) are consistent with recent U.S. EPA regulatory modeling studies (U.S. EPA 2010), and unpaired site-level peak estimation accuracy exceeds recommended performance criteria for O_3 (U.S. EPA 1991).

Supplemental Material, Table 2. 2002 network-wide $PM_{2.5}$ observations and model performance from 24-hour samples across all IMPROVE and EPA Speciation Trends Network (STN) monitoring sites in the study domain. Shown: monthly mean values of daily observed and modeled $PM_{2.5}$ and model bias ($\mu g/m^3$), fractional bias (FB, unitless) and fractional error (FE, unitless).

	IMPROVE (n = 2488, r = 0.73)					STN (n = 4777, r= 0.71)				
Month	Obs	CMAQ	Bias	FB	FE	Obs	CMAQ	Bias	FB	FE
1	7.48	12.81	5.43	0.45	0.57	13.20	20.11	7.87	0.47	0.50
2	6.61	10.53	3.96	0.40	0.47	11.07	14.73	4.26	0.32	0.42
3	7.87	10.89	3.18	0.16	0.46	11.49	12.35	1.54	0.06	0.27
4	8.09	10.36	2.57	0.16	0.39	11.36	12.86	2.15	0.12	0.34
5	9.31	10.55	1.50	0.07	0.32	10.71	11.58	1.65	0.10	0.30
6	13.94	13.94	-0.42	-0.10	0.38	17.19	16.58	0.28	-0.02	0.31
7	16.96	16.75	-0.25	-0.14	0.41	20.66	19.33	-0.61	-0.06	0.36
8	13.60	15.88	2.16	0.06	0.32	15.30	17.61	2.94	0.15	0.34
9	11.44	14.77	3.74	0.25	0.40	14.57	18.57	4.25	0.21	0.33
10	6.97	9.94	2.95	0.23	0.41	9.74	12.18	3.03	0.18	0.35
11	7.31	10.96	3.78	0.36	0.46	11.27	12.77	2.10	0.14	0.34
12	7.88	13.05	5.18	0.44	0.56	15.43	19.98	5.33	0.29	0.43
**Annual**	**9.80**	**12.59**	**2.83**	**0.20**	**0.43**	**13.66**	**15.70**	**2.70**	**0.15**	**0.36**

Supplemental Material, Table 3. 2002 JJA O_3 average observations and model performance at all hourly EPA AQS monitors in EPA Region 5 for daily maximum 8-hour and daily maximum 1-hour O_3 for days exceeding a threshold of 40 ppb (n=18062). Shown: observed and modeled O_3 and model bias and error (ppb), fractional bias (FB, unitless), fractional error (FE, unitless), r^2 (%).

Metric	Observed	CMAQ	Bias	Error	FB	FE	r^2
Daily Max 8-hour	62.95	59.60	-3.35	3.36	-0.05	0.05	98.2%
Daily Max 1-hour	67.17	67.87	0.70	9.93	0.02	0.15	51.5%

Supplemental Material, Table 4. $PM_{2.5}$ 24-hour exceedances

	MSA	Total	Rural	Suburban	Urban
Ave. change in # of NAAQS 24-hour exceedances	Chicago	0.541	0.355	0.717	1.000
	Cincinnati	0.323	0.182	0.632	0.500
	Cleveland	0.612	0.443	0.804	1.333
	Columbus	0.196	0.111	0.308	1.000
	Dayton	0.338	0.222	0.583	0.500
	Detroit	0.274	0.146	0.375	1.235
	Grand Rapids	0.257	0.259	0.269	0.000
	Indianapolis	0.262	0.205	0.400	0.571
	Madison	0.049	0.032	0.100	
	Milwaukee	0.388	0.295	0.500	0.750
	Minneapolis/St. Paul	0.093	0.077	0.136	0.154
Ave. change in exceedance amount (µg/m3)	Chicago	0.132	0.086	0.172	0.264
	Cincinnati	0.062	0.046	0.093	0.127
	Cleveland	0.079	0.051	0.114	0.185
	Columbus	0.064	0.045	0.096	0.188
	Dayton	0.053	0.044	0.068	0.130
	Detroit	0.103	0.075	0.147	0.233
	Grand Rapids	0.044	0.037	0.061	0.123
	Indianapolis	0.052	0.038	0.085	0.123
	Madison	0.081	0.080	0.084	
	Milwaukee	0.138	0.123	0.154	0.209
	Minneapolis/St. Paul	0.059	0.038	0.109	0.144
Total change in exceedances	Chicago	166	60	81	25
	Cincinnati	42	16	24	2
	Cleveland	112	51	45	16
	Columbus	28	11	12	5
	Dayton	27	12	14	1
	Detroit	75	27	27	21
	Grand Rapids	29	22	7	0
	Indianapolis	39	23	12	4
	Madison	2	1	1	
	Milwaukee	40	18	19	3
	Minneapolis/St. Paul	25	15	8	2
Max of change in exceedances	Chicago	3	3	3	2
	Cincinnati	3	2	3	2
	Cleveland	4	4	4	4
	Columbus	2	2	2	2
	Dayton	3	3	3	1
	Detroit	3	2	3	3
	Grand Rapids	2	2	2	0
	Indianapolis	2	2	2	2
	Madison	1	1	1	
	Milwaukee	2	1	2	1
	Minneapolis/St. Paul	2	2	2	1
Max of change in ave. exceedance amount	Chicago	0.332	0.286	0.332	0.332
	Cincinnati	0.197	0.197	0.197	0.197
	Cleveland	0.384	0.209	0.384	0.384
	Columbus	0.237	0.196	0.237	0.237
	Dayton	0.146	0.146	0.146	0.146
	Detroit	0.275	0.205	0.275	0.275
	Grand Rapids	0.152	0.152	0.152	0.152
	Indianapolis	0.180	0.152	0.180	0.180
	Madison	0.117	0.117	0.103	
	Milwaukee	0.288	0.247	0.288	0.288
	Minneapolis/St. Paul	0.223	0.223	0.188	0.188
Total Average of Change in exceedances		0.327	0.214	0.495	0.868
Total Average of change in average exceedance amount		0.084	0.058	0.124	0.201

Supplemental Material, Figure 1. Regional population density, 2002 estimate

Metropolitan Statistical Areas where short trips were reduced are outlined and labeled.

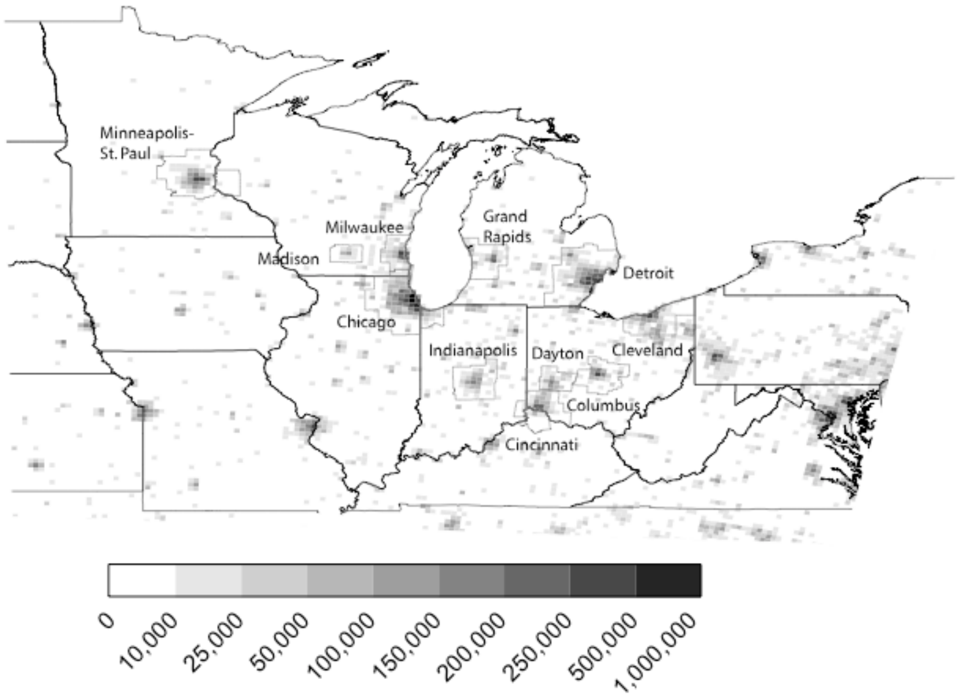

References

Boylan JW, Russell AG. 2006. PM and light extinction model performance metrics, goals, and criteria for three-dimensional air quality models. Atmos Environ. 40:4946–4959, doi:10.1016/j.atmosenv.2005.09.087.

European Cyclists Federation. 1998. Cycling in Urban Areas Position Paper. Available http://www.ecf.com/files/2/12/20/Cycling_in_urban_areas.pdf. [accessed 1 October 2009].

Morris RE, McNally DE, Tesche TW, Tonnesen G, Boylan JW, Brewer P. 2005. Preliminary evaluation of the Community Multiscale Air Quality model for 2002 over the southeastern United States. J Air Waste Manage Assoc 55:1694–1708.

Spak SN, Holloway T. 2009. Seasonality of speciated aerosol transport over the Great Lakes region. J Geophys Res 114:D08302, doi:10.1029/2008JD010598.

U.S. Census Bureau. 2008 American Community Survey. American Factfinder. http://factfinder.census.gov. [accessed 1 October 2009].

U.S. Environmental Protection Agency. 1991. Guideline for regulatory application of the urban airshed model, EPA-450/4-91-013. Research Triangle Park, N.C.

U.S. Environmental Protection Agency. 2010. Air Quality Modeling Technical Support Document: Light-Duty Vehicle Greenhouse Gas Emission Standards Final Rule. EPA 454/R-10-003. Research Triangle Park, N.C
